# Supplementary material for: One-step construction of robust protocells and prototissues in water
Source: Nat Commun. 2026 Apr 8;17:4998. doi: 10.1038/s41467-026-71650-2 (PMC13237145; doi:10.1038/s41467-026-71650-2)
Supplement: Supplementary file 1 — Supplementary Information [file 41467_2026_71650_MOESM1_ESM.pdf]

Supplementary Information

One-Step Construction of Robust Protocells and Prototissues in Water

Weixiao Feng[1,2], Peifan Li[1,2], Xin Li[1,2], Ziwei Wang[1,2], Min Chen[1,2], Yang Hu[1,3,4], Fu-Jian Xu[1,3,4*], Shaowei Shi[1,2*]

[1]State Key Laboratory of Chemical Resource Engineering, Beijing University of Chemical Technology, Beijing, 100029 China.

[2]Beijing Advanced Innovation Center for Soft Matter Science and Engineering, Beijing University of Chemical Technology, Beijing, 100029 China.

[3]Key Laboratory of Biomedical Materials of Natural Macromolecules (Beijing University of Chemical Technology), Ministry of Education, Beijing, 100029, China.

[4]Beijing Laboratory of Biomedical Materials, Beijing University of Chemical Technology, Beijing, 100029, China.

[*]E-mail: xufj@mail.buct.edu.cn; shisw@mail.buct.edu.cn

Supplementary Methods

Synthesis of fluorescently labelled dextran, CNF, enzymes and PDDA[1–5]

To synthesize FITC- or RITC-labelled dextran, 200 mg of dextran was dissolved in 20 mL of carbonate buffer (0.1 M, pH = 9.2). Under continuous stirring, 250 μL of FITC or RITC solution (1.0 mg mL^{-1} in DMSO) was slowly added to the mixture. The reaction was allowed to proceed overnight at room temperature with constant stirring.

The resulting mixture was then purified by dialysis in water for 24 h using a dialysis membrane with a molecular weight cutoff (MWCO) of 8-14 kDa. After dialysis, the product was freeze-dried to obtain FITC- or RITC-labelled dextran. Specifically, for FITC-labelled dextran with molecular weights of 10, 40, and 70 kDa, dialysis was performed using a dialysis membrane with an MWCO of 3.5 kDa to ensure effective purification. A similar procedure was employed to synthesize FITC-labelled CNF (MWCO 8–14 kDa) and FITC-labelled catalase, RITC-labelled GOx, and FITC-labelled HRP (MWCO 3.5 kDa).

For RITC labelling of PDDA, PDDA-co-DAA (200 mg) was dissolved in 20 mL of carbonate buffer (0.1 M, pH = 9.2), followed by dropwise addition of RITC (250 μL, 1.0 mg mL^{-1} in DMSO). The reaction mixture was stirred at room temperature in the dark for 24 h and purified by dialysis against deionized water (MWCO 8–14 kDa) for 48 h, followed by lyophilization to yield PDDA-RITC.

Synthesis of GO-Pyrene[6]

Graphene oxide (GO, 100 mg) was refluxed in thionyl chloride (50 mL) for 12 h, and excess thionyl chloride was removed by rotary evaporation to afford GO–COCl (91 mg). GO–COCl was then reacted with cysteine (50 mg) in DMF (50 mL) in the presence of triethylamine (0.5 mL) at room temperature for 24 h to yield GO–Cys (80 mg) after filtration, washing (DMF, deionized water, and ethanol), and vacuum drying. GO–Cys was dispersed in DMF/H_2O (50 mL, v/v ≈ 4:1), treated with TCEP (50 mg) for 12 h, and then reacted with pyrene–maleimide (50 mg) at room temperature for 24

h. The product was collected and washed with DMF, deionized water, and ethanol, followed by repeated ultrasonication in fresh ethanol to remove physically adsorbed pyrene–maleimide, and finally vacuum-dried to afford GO–Pyrene (52 mg).

In Vitro Cytotoxicity Assay

L929 cells were cultured in complete DMEM supplemented with 10% fetal bovine serum (FBS) and 1% penicillin–streptomycin at 37 °C in a humidified incubator with 5% CO_2. For extract preparation, freeze-dried CNF-GO/PDDA protocells prepared with different surfactants were incubated in complete DMEM at 4 mg mL^{-1} for 24 h and the extracts were sterilized by filtration. L929 cells were seeded in 96-well plates at a density of 5,000 cells per well (n = 3 wells per group) and allowed to adhere for 24 h, after which the medium was replaced with the corresponding extracts; the blank control group received fresh complete DMEM. Cells were cultured for 3 days, and the corresponding fresh extracts were replaced daily. For the CCK-8 assay, CCK-8 reagent was added to each well at 10% (v/v) of the culture medium and incubated for 1 h, and the absorbance at 450 nm was measured using a microplate reader. At the end of the culture period, the medium was removed, cells were rinsed with 1 × Assay Buffer, and live/dead staining was performed by incubating with Calcein-AM (2 μM) at 37 °C for 25 min in the dark followed by PI (4 μM) at 37 °C for 5 min in the dark; fluorescence images were acquired to visualize live (green) and dead (red) cells. Data are presented as mean ± standard deviation, and statistical significance among groups was evaluated by one-way ANOVA with Tukey's post hoc test.

Supplementary Figures

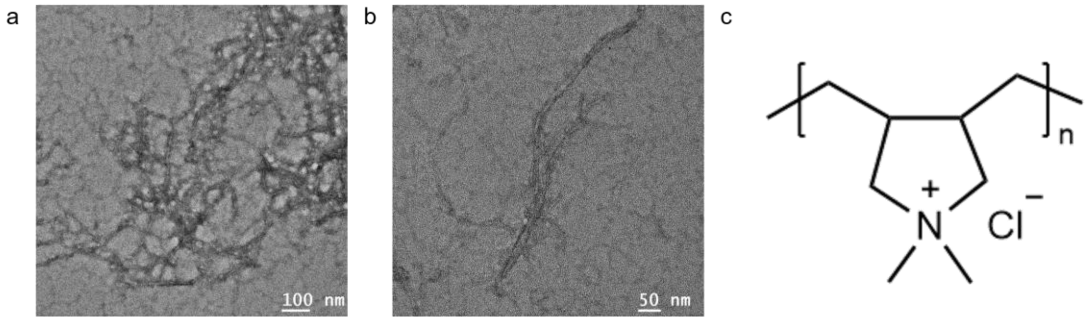

Supplementary Fig. 1: Morphology of CNF and chemical structure of PDDA. a,b TEM images of CNF. **c** Chemical structure of PDDA. Experiments were repeated independently 3 times with similar results.

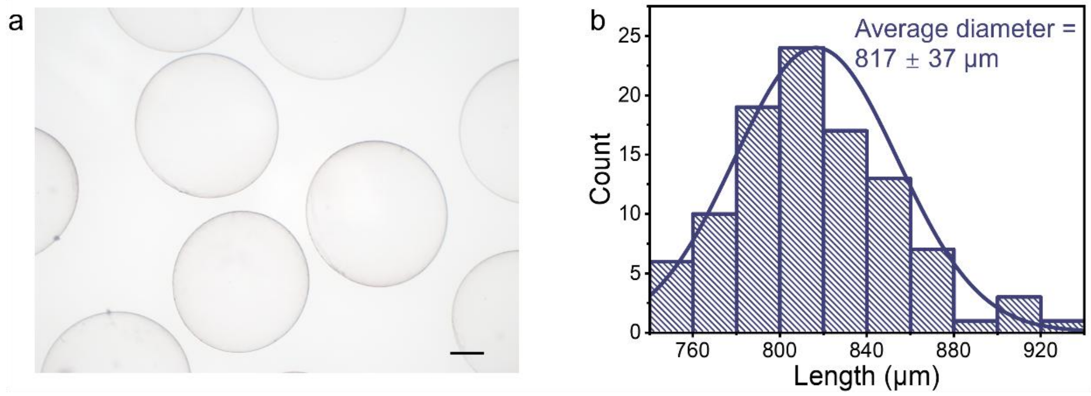

Supplementary Fig. 2: Optical image and size distribution of CNF/PDDA microcapsules. a, Optical microscopy image of CNF/PDDA microcapsules. **b**, Size distribution of CNF/PDDA microcapsules. Data are presented as mean values ± standard deviation (n = 100 microcapsules). Scale bar, 200 μm. Source data are provided as a Source Data file.

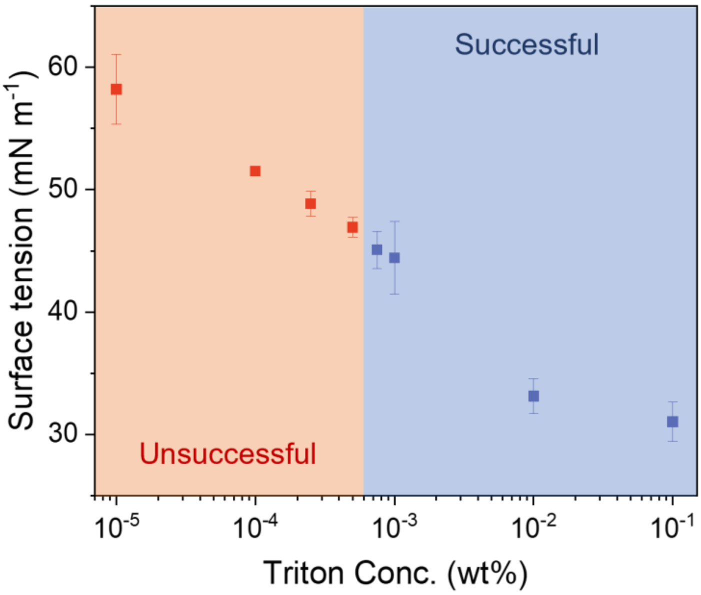

Supplementary Fig. 3: Effect of Triton X-100 concentration on air-water surface tension and microcapsule formation. Source data are provided as a Source Data file.

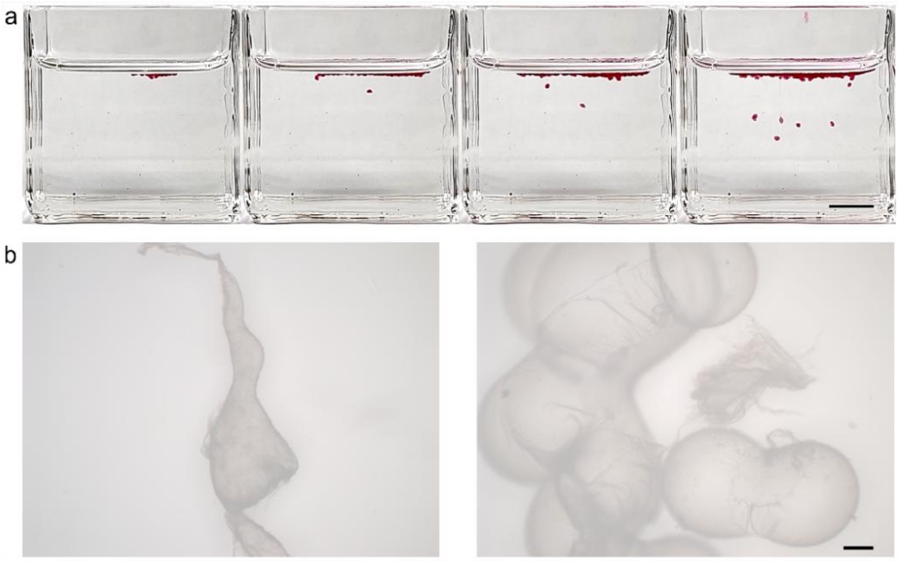

Supplementary Fig. 4: Failed fabrication of CNF/PDDA microcapsules without Triton X-100. a, Digital images showing the unsuccessful fabrication of CNF/PDDA microcapsules via gas–liquid microfluidic-assisted DIC, where the extruded phase was dyed with Amaranth Red for enhanced visualization and Triton X-100 was not added to the aqueous collection bath. Scale bar, 1 cm. **b**, Optical microscopy images of irregularly shaped microcapsules and aggregate structures prepared without Triton X-

100. Scale bar, 200 μm. Experiments were repeated independently 3 times with similar results.

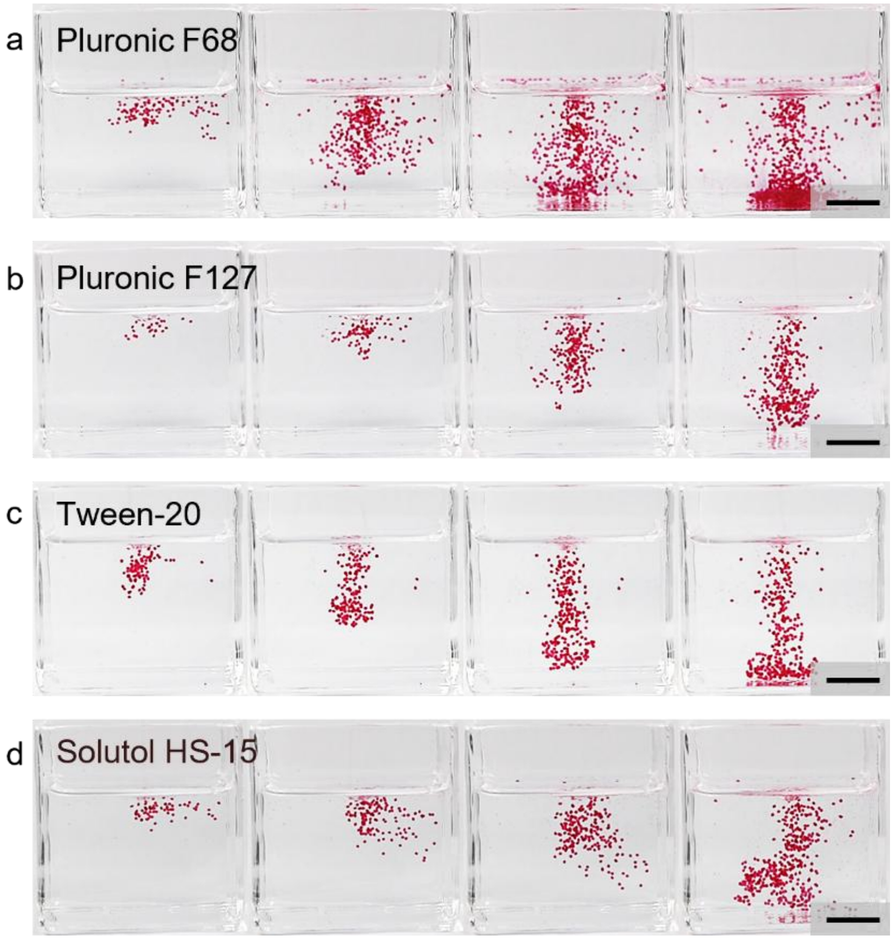

Supplementary Fig. 5: Fabrication of CNF/PDDA microcapsules using different surfactants. a-d, Digital images showing the successful fabrication of CNF/PDDA microcapsules using four different surfactants including Pluronic F68, Pluronic F127, Tween-20, and Solutol HS-15. [CNF] = 0.6 wt%; [PDDA] = 0.5 wt%; [Surfactant] = 0.1 wt%; extrusion rate = 30 mL h^{-1}; gas flow rate = 1.0 L min^{-1}. Scale bar, 1 cm. Experiments were repeated independently 3 times with similar results.

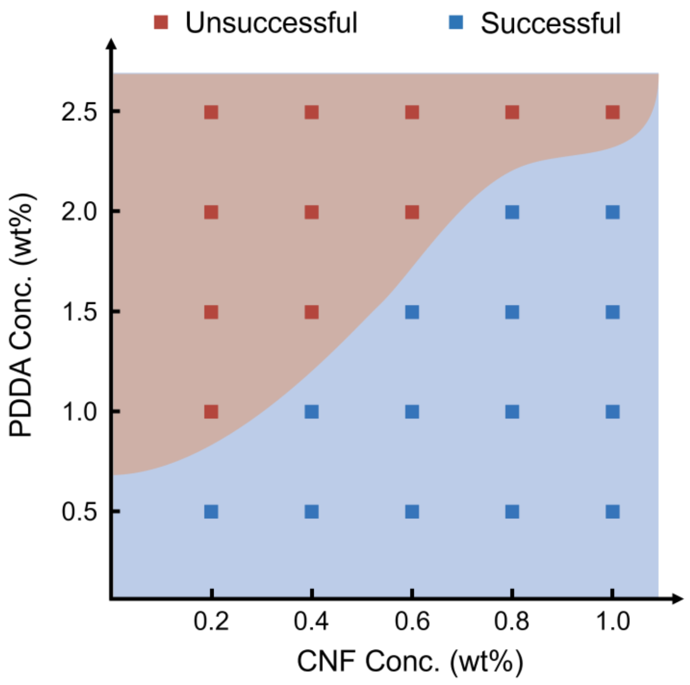

Supplementary Fig. 6: Effects of CNF and PDDA concentrations on microcapsule formation. [Triton X-100] = 0.1 wt%; extrusion rate = 30 mL h^{-1}; gas flow rate = 1.0 L min^{-1}.

When the CNF concentration was fixed, increasing the PDDA concentration raised the viscosity of the collection bath, which prevented CNF-containing droplets from entering the bath smoothly and thus hindered microcapsule formation. On the other hand, at a fixed PDDA concentration, increasing the CNF concentration enhanced the mechanical strength of microcapsules, thereby facilitating their formation. Within the concentration range that yielded microcapsules, the size remained nearly constant at ~ 800 μm, indicating that concentration was not the key factor governing microcapsule dimensions.

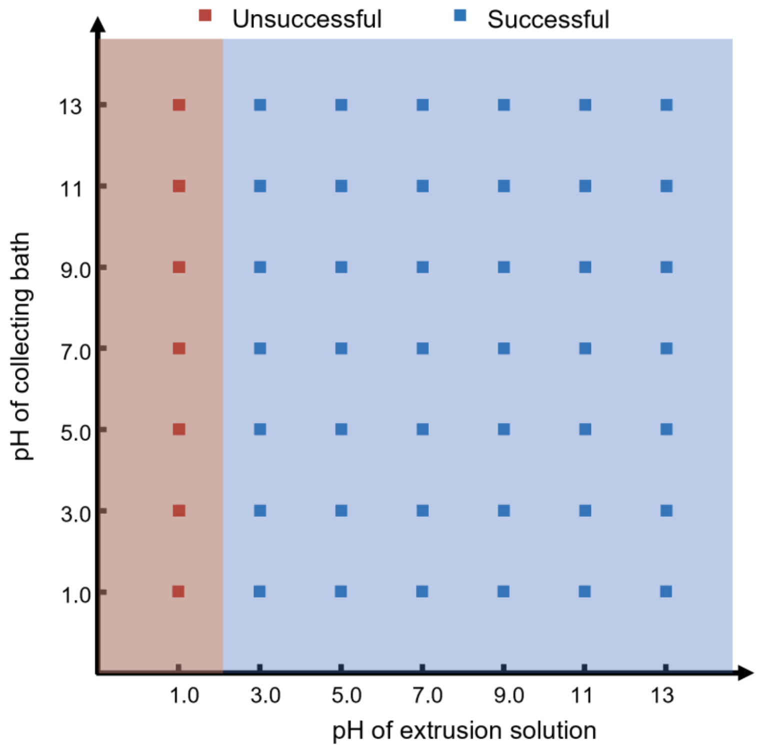

Supplementary Fig. 7: Effect of pH on microcapsule formation. [CNF] = 0.6 wt%; [PDDA] = 0.5 wt%; [Triton X-100] = 0.1 wt%; extrusion rate = 30 mL h^{-1}; gas flow rate = 1.0 L min^{-1}.

Microcapsules could be successfully produced over a wide pH range. At extremely low pH (pH = 1.0), however, extensive protonation of the carboxyl groups on CNF weakened electrostatic interactions with PDDA, resulting in failure of microcapsule formation. Within the pH range that yielded microcapsules, the capsule size remained nearly constant at ~ 800 μm, indicating that pH was not the key factor governing microcapsule dimensions. In our work, we did not intentionally adjust the pH of either solution. The 0.6 wt% CNF and 0.5 wt% PDDA solutions were used as prepared, with measured pH values of approximately 7.0 and 5.0, respectively.

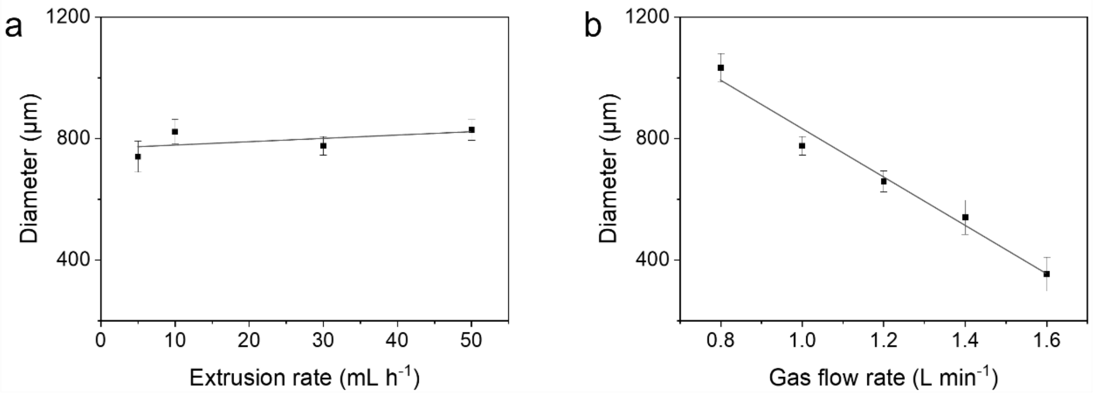

Supplementary Fig. 8: Microcapsule size as a function of extrusion rate and gas flow rate. Plots of the diameter of microcapsules prepared at different extrusion rates (**a**, gas flow rate was fixed at 1.0 L min^{-1}), and different gas flow rates (**b**, extrusion rate was fixed at 30 mL h^{-1}). [CNF] = 0.6 wt%; [PDDA] = 0.5 wt%; [Triton X-100] = 0.1 wt%; Data are presented as mean values ± standard deviation (n = 50 microcapsules). Source data are provided as a Source Data file.

At a fixed gas flow rate of 1.0 L min^{-1}, varying the extrusion rate from 5.0 to 50 mL h^{-1} produced microcapsules with nearly constant sizes of ~ 800 µm. In contrast, at a fixed extrusion rate of 30 mL h^{-1}, increasing the gas flow rate from 0.8 to 1.6 L min^{-1} reduced the microcapsule size from ~1000 to ~ 400 µm. These results indicated that microcapsule size was governed primarily by the gas flow rate rather than the extrusion rate.

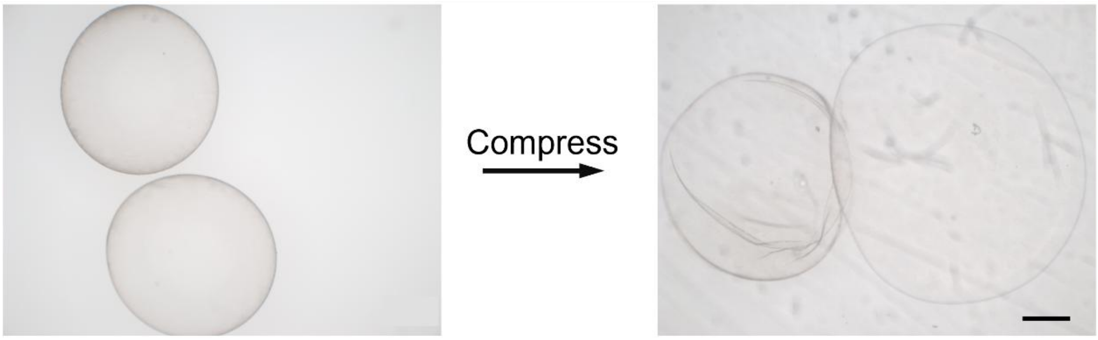

Supplementary Fig. 9: Mechanical stability of CNF/PDDA microcapsules under compression. Scale bar, 200 μm. Experiments were repeated independently 3 times with similar results.

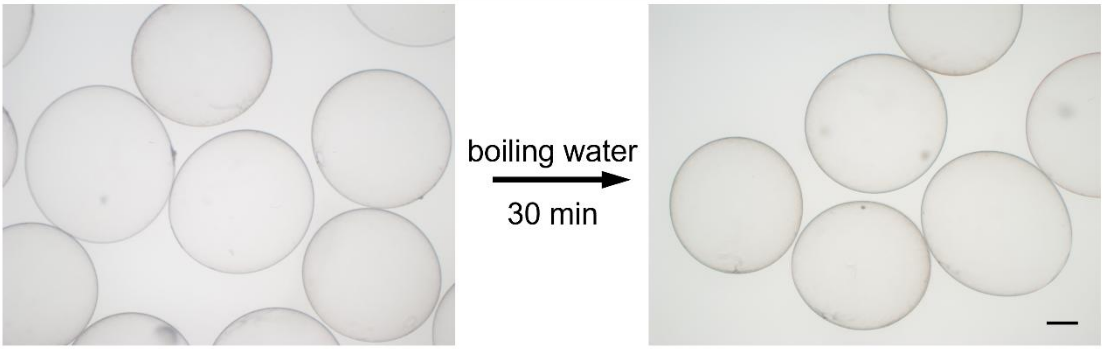

Supplementary Fig. 10: Thermal stability of CNF/PDDA microcapsules in boiling water. Optical microscopy images showing the thermal stability of CNF/PDDA microcapsules in boiling water. Scale bar, 200 μm. Experiments were repeated independently 3 times with similar results.

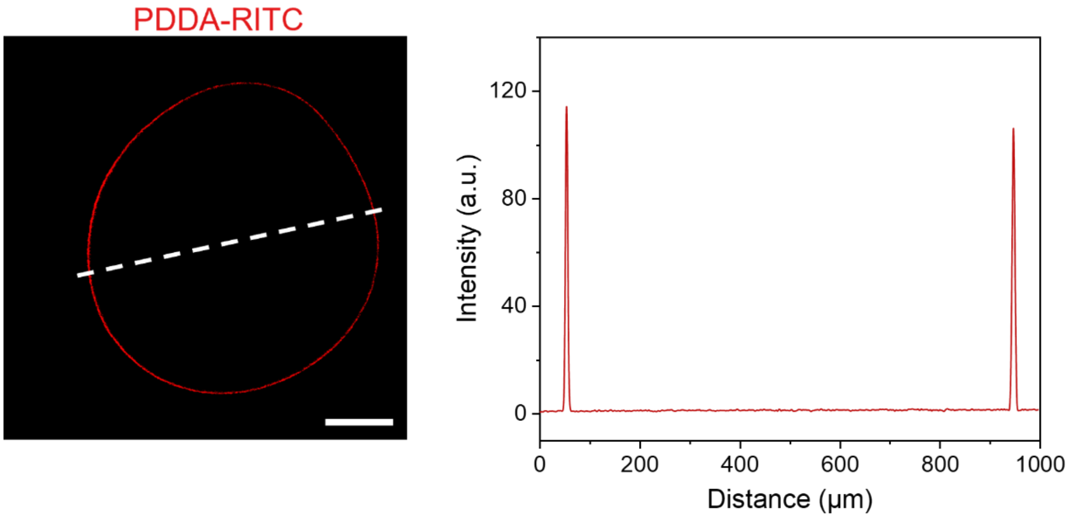

Supplementary Fig. 11: Spatial distribution of PDDA in CNF/PDDA microcapsules. CLSM images of a CNF/PDDA microcapsule containing PDDA-RITC and corresponding fluorescence intensity profile along the dashed line. Scale bar, 200

μm. Source data are provided as a Source Data file. Experiments were repeated independently 3 times with similar results.

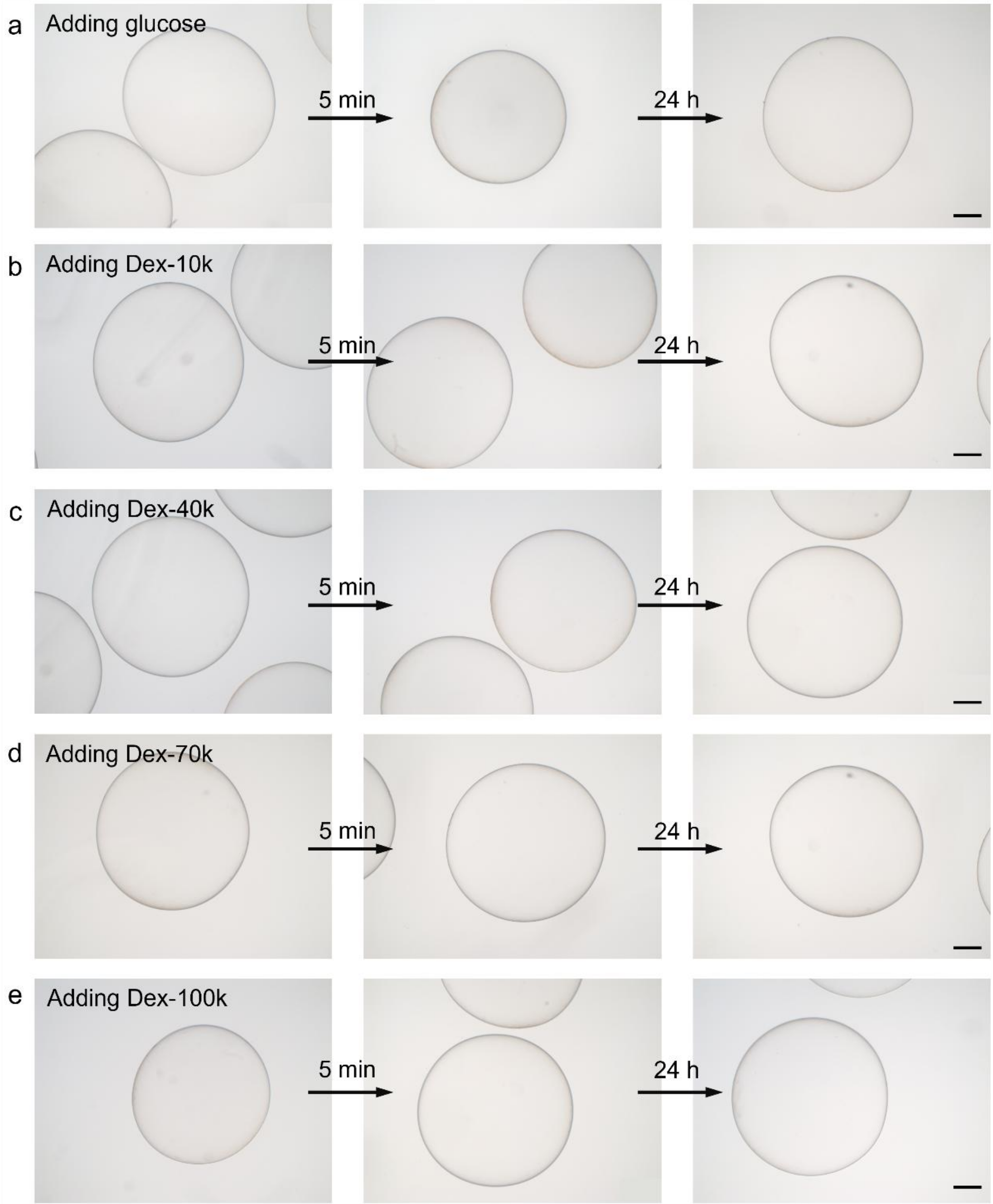

Supplementary Fig. 12: Osmotic tests of CNF/PDDA microcapsules in dextran solutions. a-e, Optical microscopy images showing the morphological evolution of CNF/PDDA microcapsules aged in hypertonic solutions of 1 M glucose (**a**), 1 wt%

Dex-10k (**b**), 1 wt% Dex-40k (**c**), 1 wt% Dex-70k (**d**), and 1 wt% Dex-100k (**e**). Scale bar, 200 µm. Experiments were repeated independently 3 times with similar results.

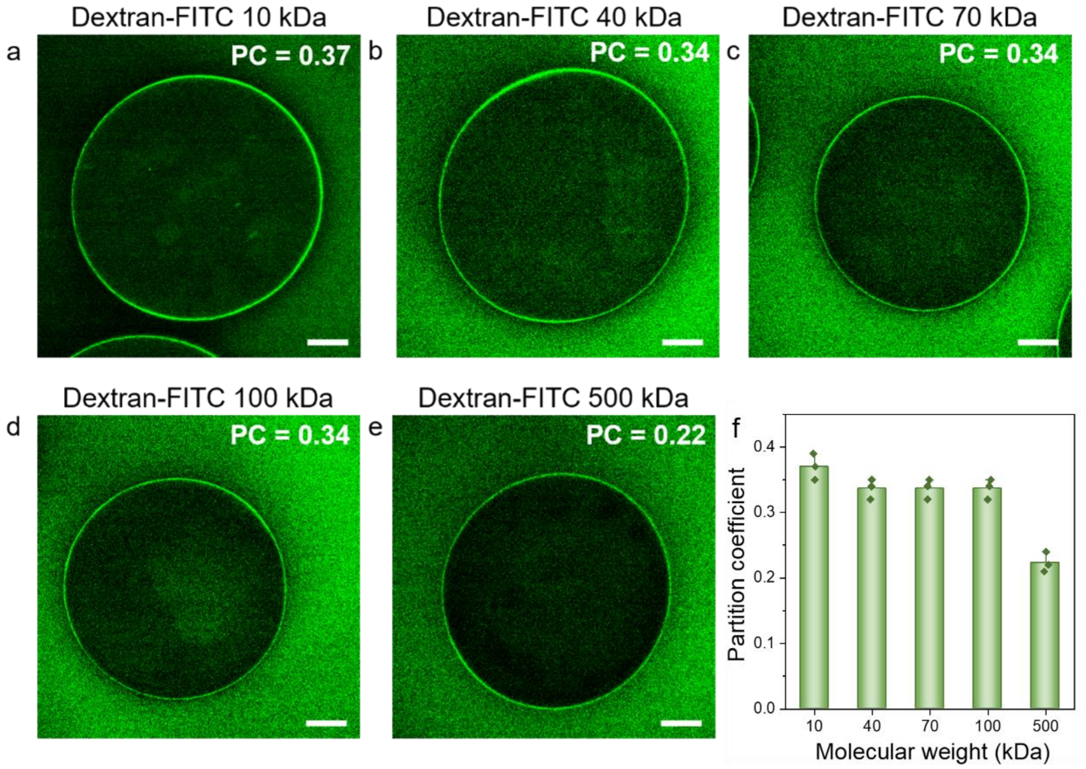

Supplementary Fig. 13: Partitioning of Dextran in CNF/PDDA microcapsules. a-e, Confocal fluorescence images of CNF/PDDA microcapsules after equilibration for 4 h in dextran solutions containing trace amounts of FITC-dextran. Scale bars, 200 µm. **f,** Summary of partition coefficients (PCs) of dextran, determined using FITC-dextran as a fluorescent tracer, where the PC is defined as the ratio of internal to external fluorescence intensity. Data are presented as mean values ± standard deviation (n = 3 independent experiments). Source data are provided as a Source Data file.

Since the concentration of Dex-FITC inside and outside the CNF/PDDA microcapsules was proportional to the fluorescence intensity, the partition coefficient (PC) could be determined from the ratio of internal to external fluorescence intensity. Using PC = $C_{in}/C_{out} \approx I_{in}/I_{out}$, the PCs of Dex-FITC with molecular weights of 10, 40, 70, 100, and 500 kDa were 0.37, 0.34, 0.34, 0.34, and 0.22, respectively. It should be

noted that, although the CNF/PDDA microcapsule membrane did not block Dex-FITC, the interior was filled with a loose CNF network that reduced the accessible free volume for dextran. In addition, negatively charged CNF introduced electrostatic repulsion against the anionic FITC label. Both steric exclusion and electrostatic effects led to a lower Dex-FITC content inside the microcapsules compared to the exterior.

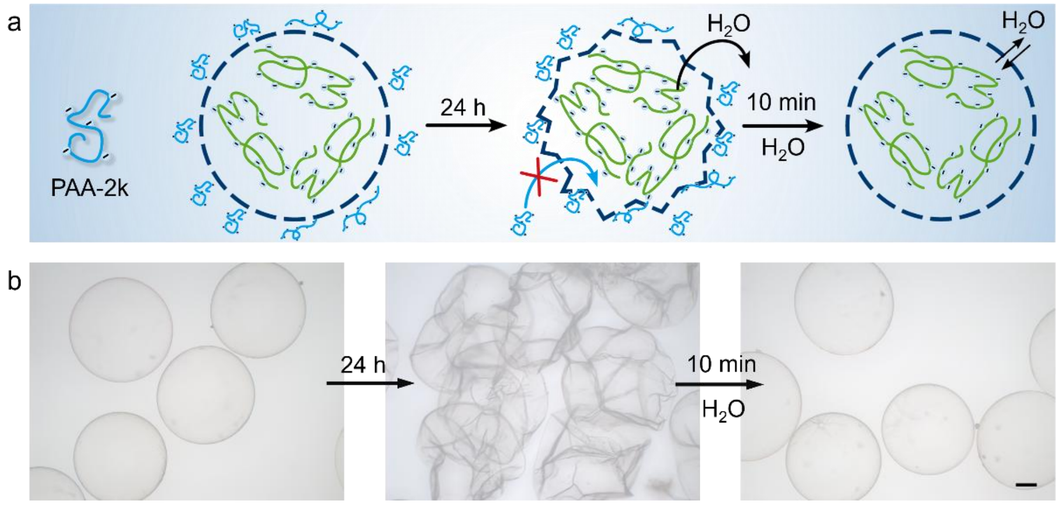

Supplementary Fig. 14: Reversible shape change of CNF/PDDA microcapsules induced by PAA-2k. a,b, Schematic illustration (**a**) and optical microscopy images (**b**) showing the morphological evolution of CNF/PDDA microcapsules first aged in a hypertonic solution of 1 wt% PAA-2k and subsequently in pure water. Scale bar, 200 μm. Experiments were repeated independently 3 times with similar results.

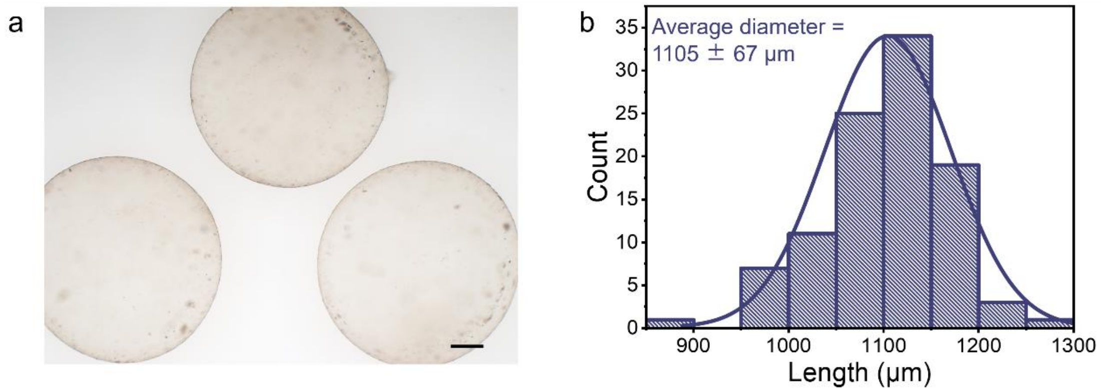

Supplementary Fig. 15: Morphology and size distribution of CNF-GO/PDDA microcapsules. a, Optical microscopy image of CNF-GO/PDDA microcapsules. **b**, Size distribution of CNF-GO/PDDA microcapsules. Data are presented as mean values ± standard deviation (n = 100 microcapsules). Scale bar, 200 μm. Source data are provided as a Source Data file.

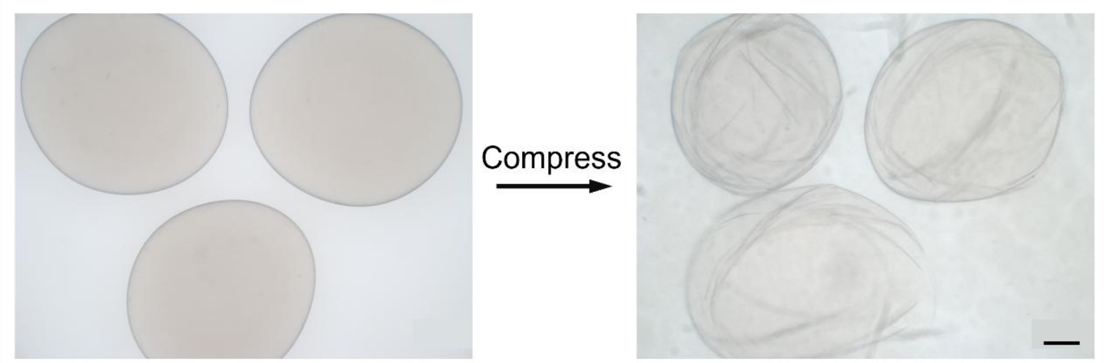

Supplementary Fig. 16: Mechanical stability of CNF-GO/PDDA microcapsules under compression. Scale bar, 200 μm. Experiments were repeated independently 3 times with similar results.

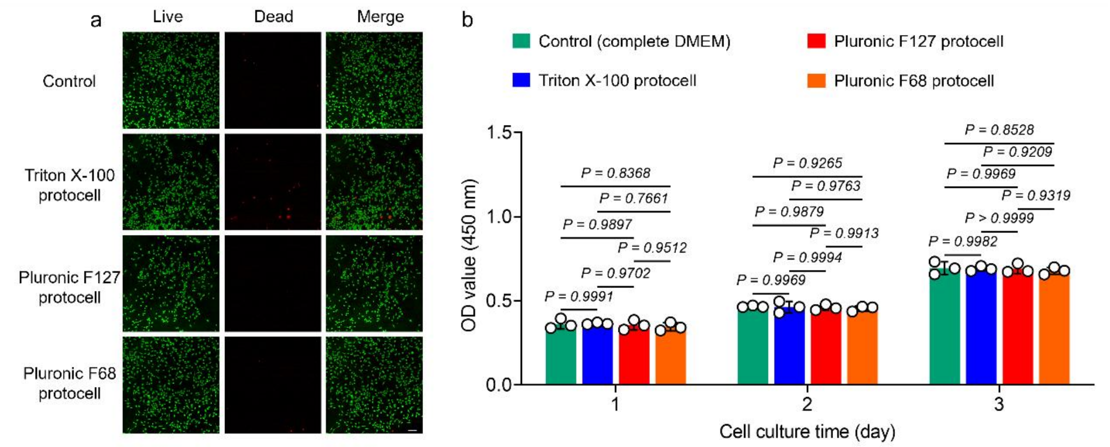

Supplementary Fig. 17: Cytocompatibility of CNF-GO/PDDA protocells. a, Live/dead staining images of L929 cells after incubation with the control medium or with Triton X-100 protocells, Pluronic F127 protocells, and Pluronic F68 protocells. Live cells are stained green with Calcein-AM and dead cells are stained red with PI,

with merged images shown in the right column. Scale bar, 100 µm. **b**, CCK-8 assay results (absorbance at 450 nm) of L929 cells cultured with the corresponding extracts for 1–3 days. Data are presented as mean values ± standard deviation (n = 3 wells per group), and P values are indicated. Source data are provided as a Source Data file.

L929 cells incubated with extracts from CNF-GO/PDDA protocells prepared at a low surfactant concentration exhibited predominantly live (green) cells with few dead (red) cells in the live/dead staining images, and the cell morphology was comparable to that of the control group. Consistently, the CCK-8 assay showed no obvious differences in cell viability between the extract-treated groups and the control over 1–3 days. These results indicate good cytocompatibility of CNF-GO/PDDA protocells prepared under low-surfactant conditions.

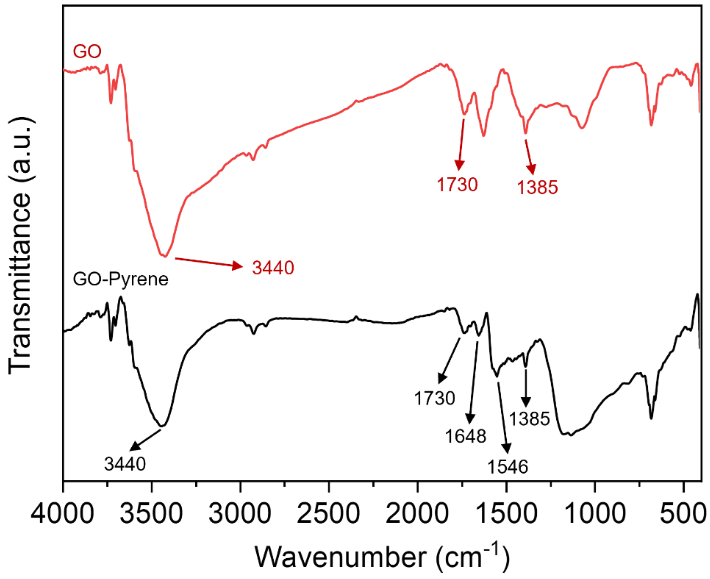

Supplementary Fig. 18: FTIR characterization of GO-pyrene. FTIR spectra of pristine GO (red) and GO–pyrene (black). Source data are provided as a Source Data file.

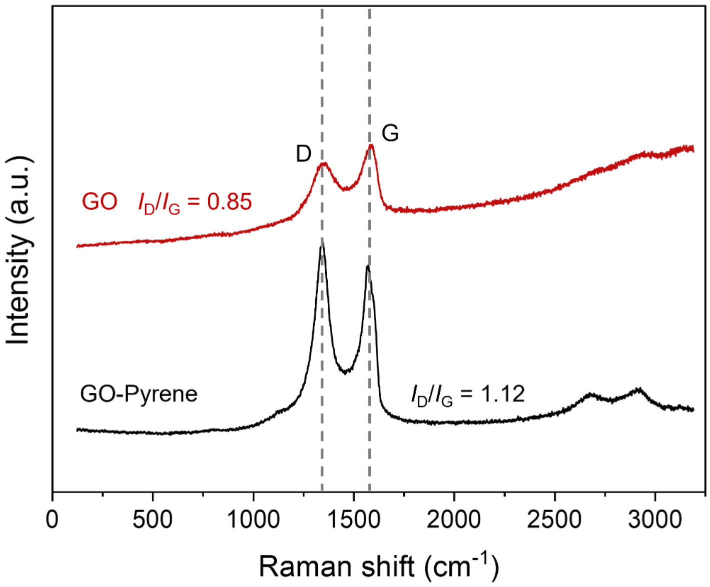

Supplementary Fig. 19: Raman characterization of GO-pyrene. Raman spectra of pristine GO (red) and GO–pyrene (black) (excitation: 514 nm). Source data are provided as a Source Data file.

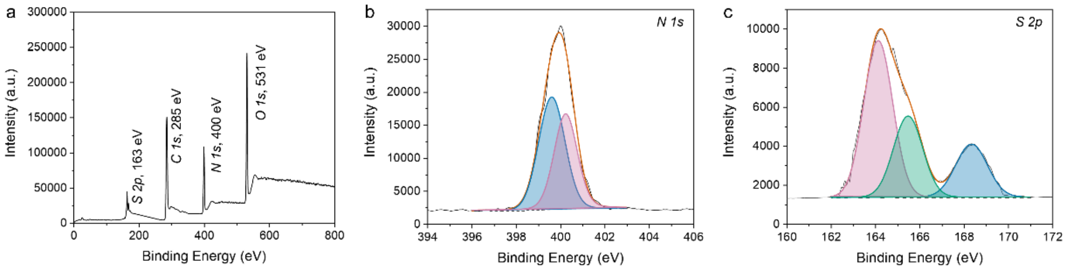

Supplementary Fig. 20: XPS characterization of GO-pyrene. XPS analysis of GO–Pyrene. **a**, Survey spectrum of GO–Pyrene. **b**, High-resolution *N 1s* spectrum. **c**, High-resolution *S 2p* spectrum. Source data are provided as a Source Data file.

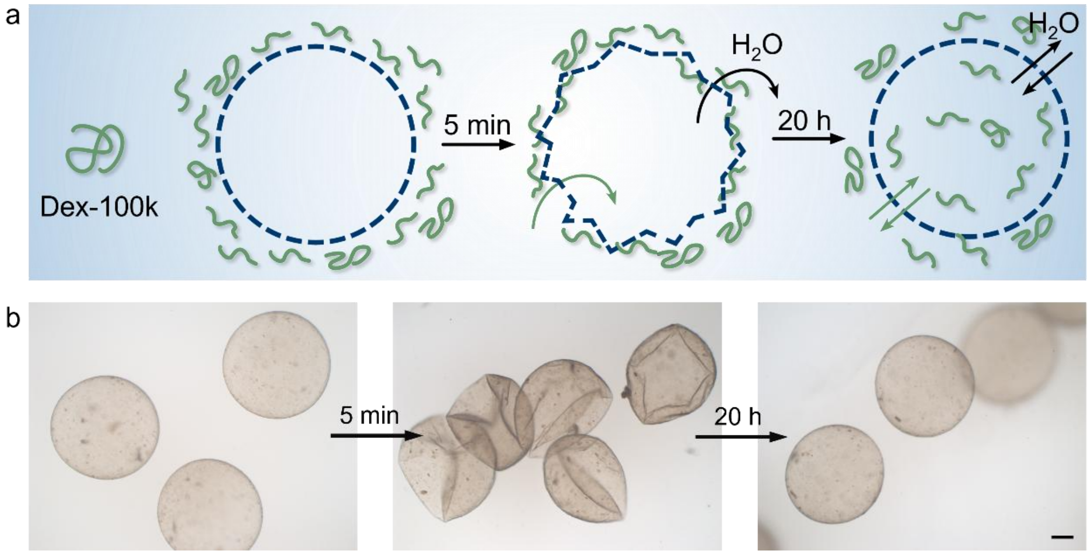

Supplementary Fig. 21: Osmotic deformation of CNF-GO/PDDA microcapsules in Dex-100k solution. a,b, Schematic illustration (**a**) and optical microscopy images (**b**) showing the morphological evolution of CNF-GO/PDDA microcapsules aged in a hypertonic solution of 1 wt% Dex-100k. Scale bar, 200 μm. Experiments were repeated independently 3 times with similar results.

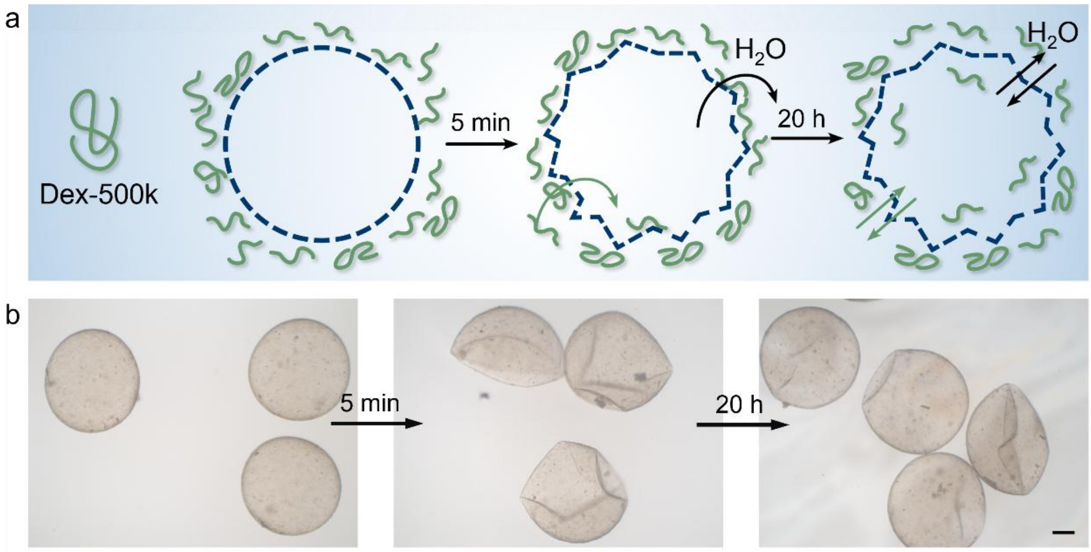

Supplementary Fig. 22: Osmotic deformation of CNF-GO/PDDA microcapsules in Dex-500k solution. a,b, Schematic illustration (**a**) and optical microscopy images (**b**) showing the morphological evolution of CNF-GO/PDDA microcapsules aged in a

hypertonic solution of 1 wt% Dex-500k. Scale bar, 200 μm. Experiments were repeated independently 3 times with similar results.

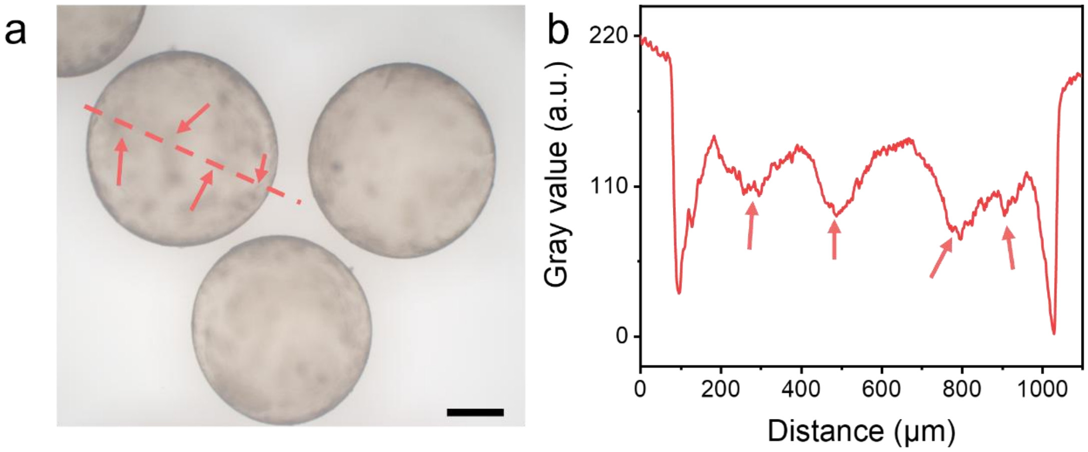

Supplementary Fig. 23: Formation of dark microdomains at the surface of CNF-GO/PDDA microcapsules. a,b, Optical microscopy images (**a**) showing the formation of dark microdomains at the surface of CNF-GO/PDDA microcapsules, and the corresponding gray value profile (**b**) along the dashed line in (**a**). Scale bar, 200 μm. Source data are provided as a Source Data file. Experiments were repeated independently 3 times with similar results.

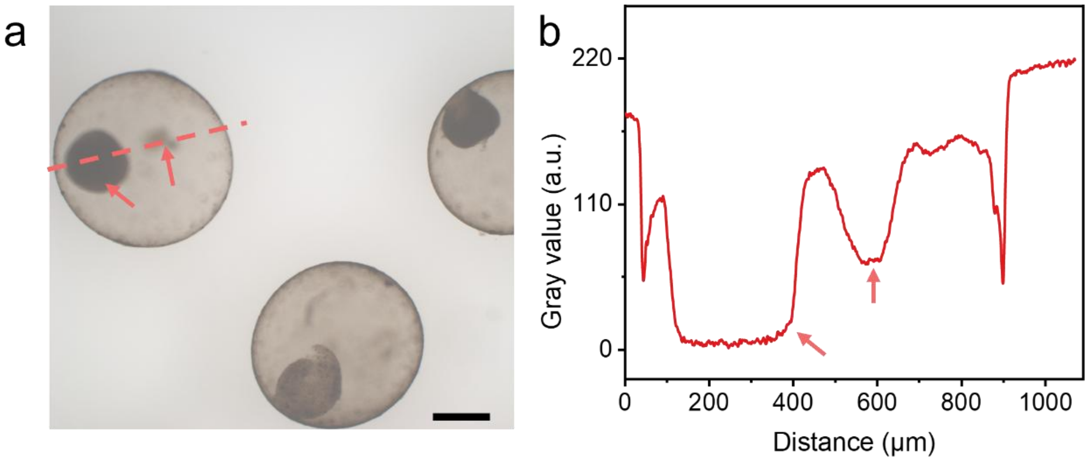

Supplementary Fig. 24: Formation of dark aggregates inside the CNF-GO/PDDA microcapsules. a,b, Optical microscopy images (**a**) showing the formation of dark aggregates inside the CNF-GO/PDDA microcapsules, and the corresponding gray value profile (**b**) along the dashed line in (**a**). Scale bar, 200 µm. Source data are provided as a Source Data file. Experiments were repeated independently 3 times with similar results.

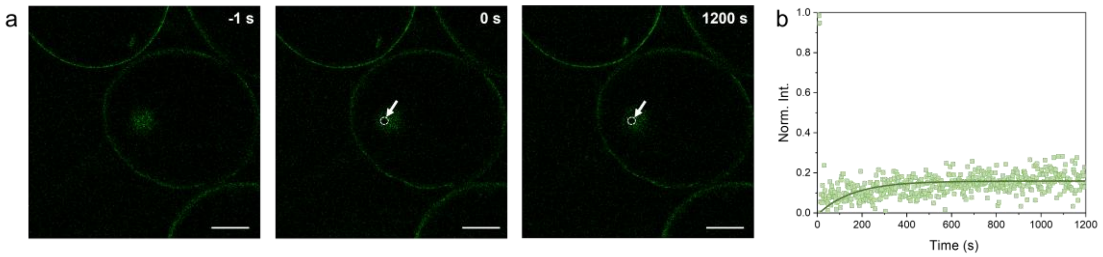

Supplementary Fig. 25: FRAP analysis of PEI-FITC in CNF-GO/PDDA microcapsules. a, Confocal fluorescence images of a CNF–GO/PDDA microcapsule containing PEI–FITC recorded before bleaching (-1 s), immediately after bleaching (0 s) and after 1200 s recovery, with the bleached region indicated by an arrow. Scale bar, 200 µm. **b,** Corresponding fluorescence intensity in the bleached region. Source data are provided as a Source Data file. Experiments were repeated independently 3 times with similar results.

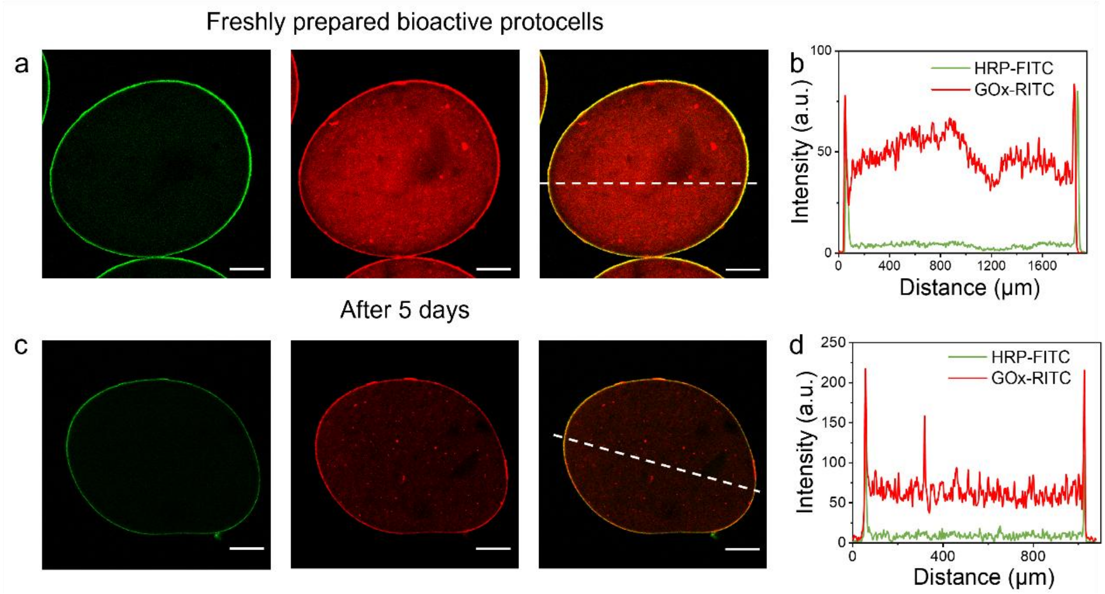

Supplementary Fig. 26: Encapsulation stability of enzymes in CNF-GO/PDDA protocells. a, CLSM images of freshly prepared CNF-GO/PDDA protocells loaded with HRP-FITC and GOx-RITC. Scale bar, 200 μm. **b**, Fluorescence intensity profiles along the white dashed line in (a). **c**, CLSM images of CNF-GO/PDDA protocells after aging for 5 days in water. Scale bar, 200 μm. **d**, Fluorescence intensity profiles along the white dashed line in (c). Source data are provided as a Source Data file. Experiments were repeated independently 3 times with similar results.

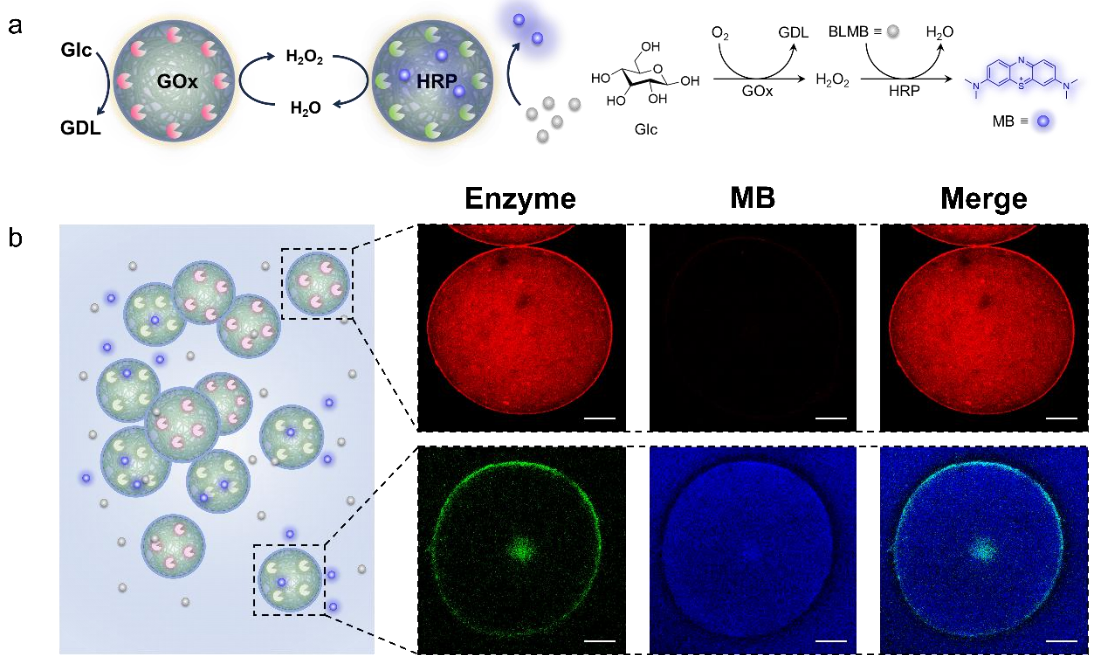

Supplementary Fig. 27: Schematic and experimental demonstration of chemical signaling between enzyme-loaded protocells. **a**, Schematic illustration of the chemical signaling between single-enzyme-loaded protocells. **b**, Schematic illustration and CLSM images showing the differences in chemical reactions within protocells loaded with different enzymes. Scale bar, 200 μm. Experiments were repeated independently 3 times with similar results.

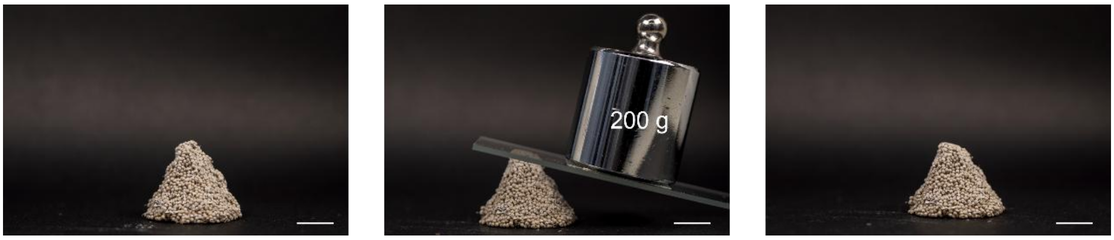

Supplementary Fig. 28: Mechanical stability of freeze-dried CNF-GO/PDDA prototissues. Digital images showing the excellent mechanical stability of freeze-dried CNF–GO/PDDA prototissue. Scale bar, 1 cm. Experiments were repeated independently 3 times with similar results.

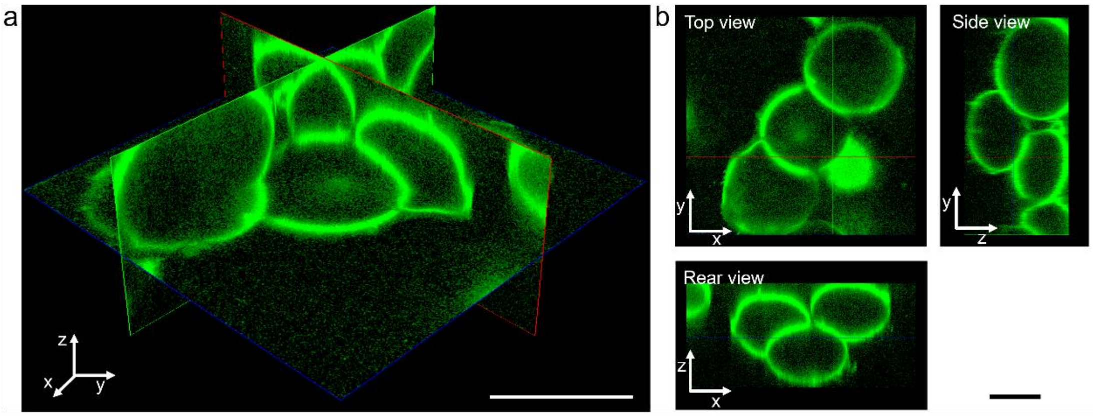

Supplementary Fig. 29: Three-dimensional CLSM reconstruction of a CNF-GO/PDDA prototissue. a,**b**, Clipping analysis for a 3D reconstructed CLSM image of a CNF-GO/PDDA prototissue containing CNF-FITC. Scale bar, 500 µm. Experiments were repeated independently 3 times with similar results.

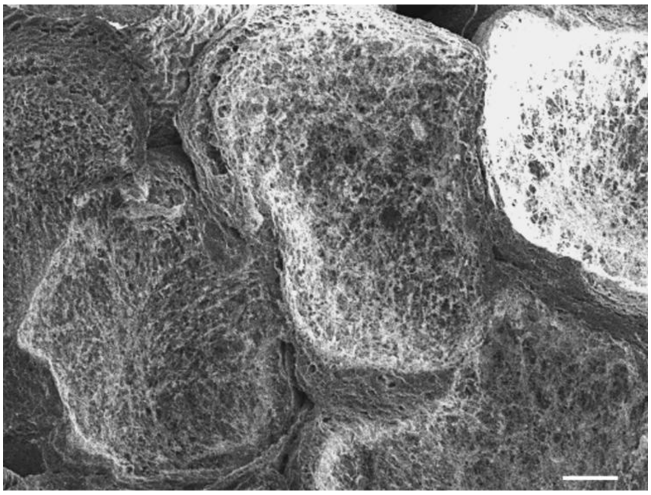

Supplementary Fig. 30: Cross-sectional morphology of a freeze-dried prototissue. SEM image of the cross-section of a freeze-dried prototissue. Scale bar, 100 µm. Experiments were repeated independently 3 times with similar results.

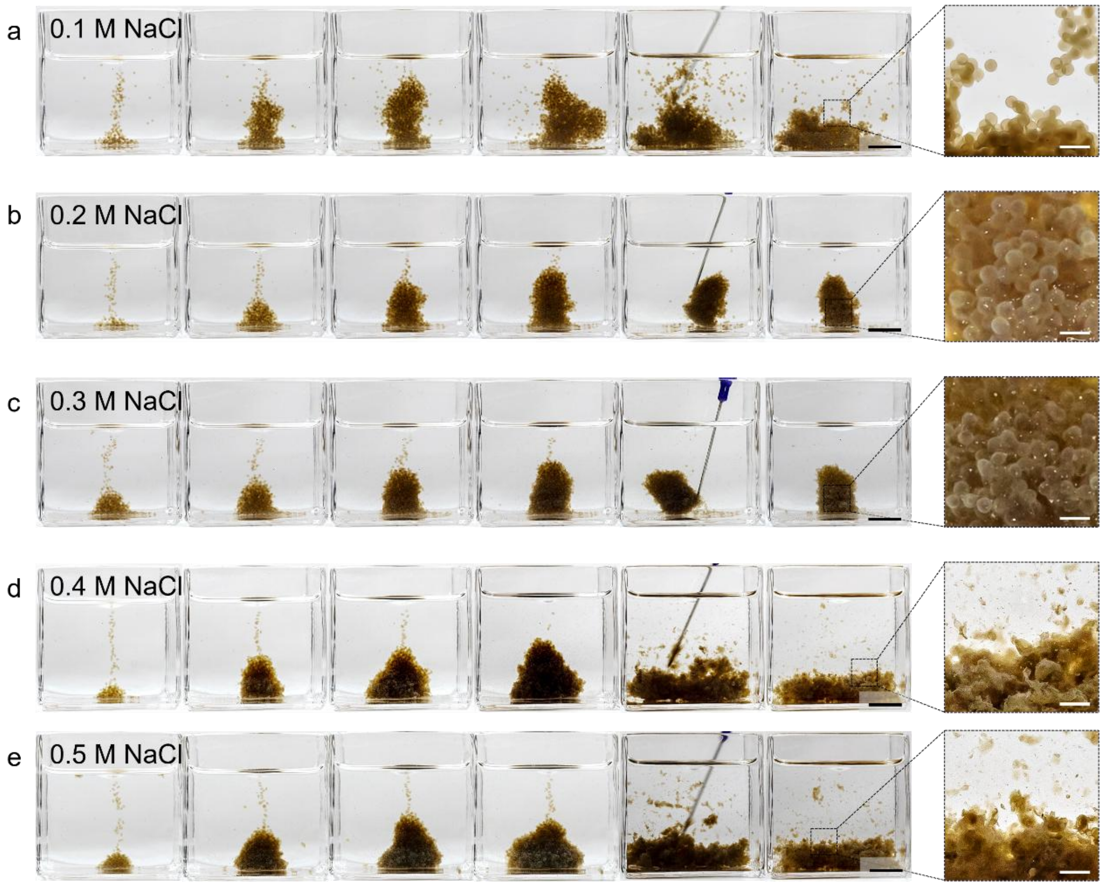

Supplementary Fig. 31: Effect of ionic strength on CNF-GO/PDDA prototissue construction. Influence of ionic strength on the construction of CNF-GO/PDDA prototissues by adding NaCl at 0.1 M (**a**), 0.2 M (**b**), 0.3 M (**c**), 0.4 M (**d**), and 0.5 M (**e**) into the extrusion droplets. Scale bar, 1 cm (left), 0.2 cm (right). Experiments were repeated independently 3 times with similar results.

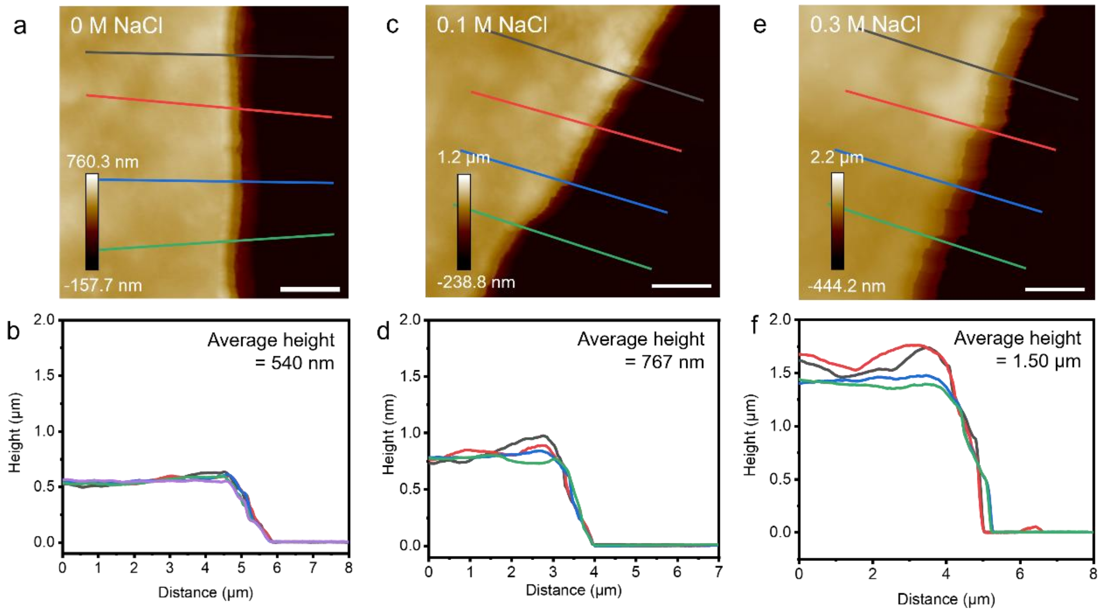

Supplementary Fig. 32: AFM characterization of 2D CNF-GO/PDDA membranes prepared with different NaCl concentrations. a-f, AFM images (**a,c,e**) of 2D CNF-GO/PDDA membranes prepared at different concentrations of NaCl and corresponding line cut analysis (**b,d,f**) of the film edge in (**a,c,e**). Scale bar, 2.0 μm. Source data are provided as a Source Data file. Experiments were repeated independently 3 times with similar results.

The 2D film samples for AFM characterization were prepared via a layer-by-layer assembly approach. First, an appropriate amount of CNF-GO aqueous dispersion containing different concentrations of NaCl was added to the bottom of a clean Petri dish. Subsequently, a small volume of PDDA aqueous solution was gently introduced on top of the CNF-GO dispersion. After allowing 5 min for film assembly, the upper and lower surfaces of the film were thoroughly rinsed with a large volume of deionized water to remove residual CNF, GO, and PDDA. Finally, the film was transferred onto a silicon wafer that had been pre-cleaned by ultrasonic treatment in acetone, and the film thickness was characterized by AFM.

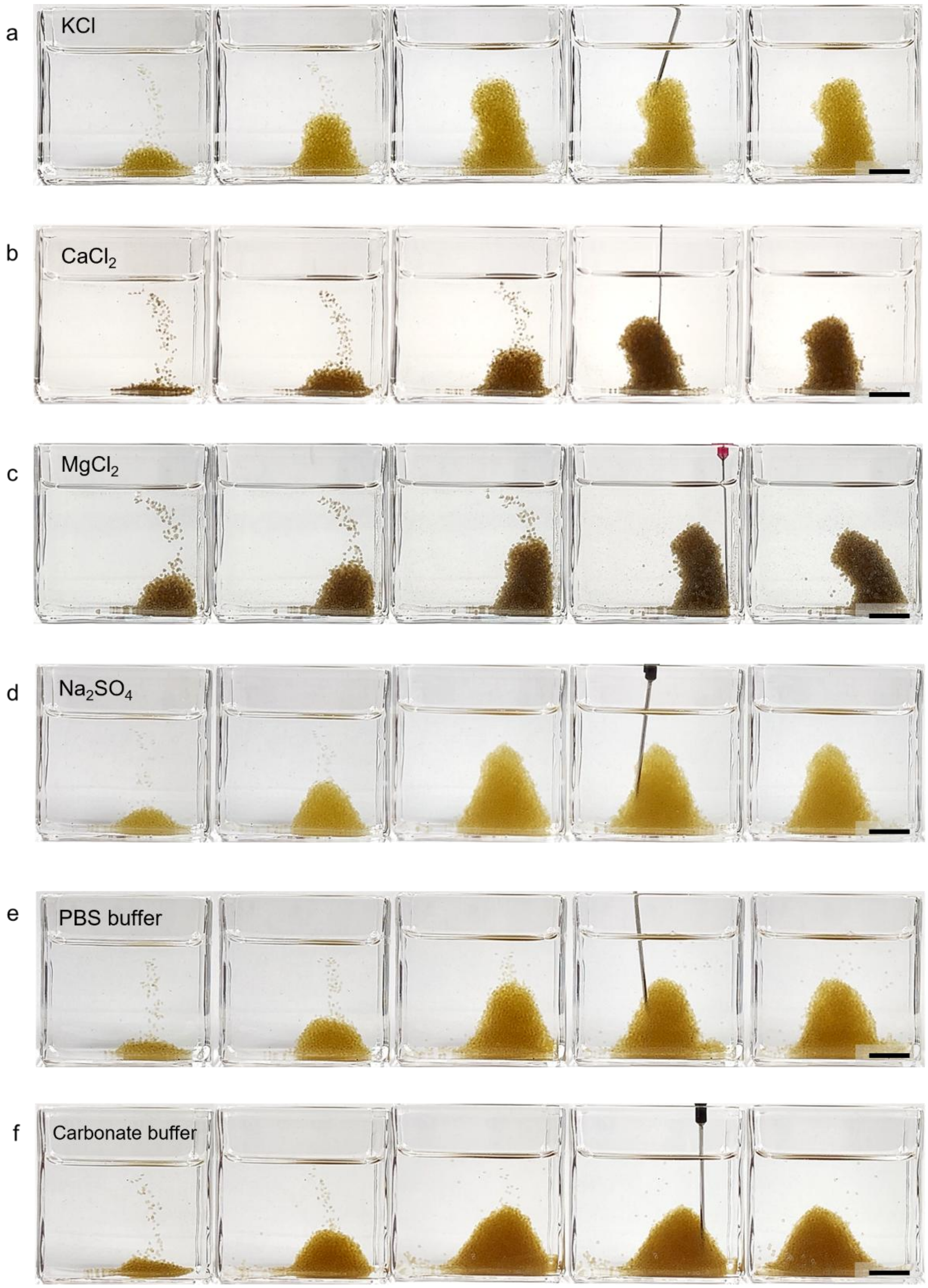

Supplementary Fig. 33: Construction of CNF-GO/PDDA prototissues with different salts and buffers. a-f, Digital images showing the construction of CNF-GO/PDDA prototissues via gas-liquid microfluidic-assisted DIC by adding 0.3 M KCl (**a**), 0.15 M $CaCl_2$ (**b**), 0.15 M $MgCl_2$ (**c**), 0.15 M Na_2SO_4 (**d**), 0.1 M PBS buffer (**e**), and 0.1 M carbonate buffer (**f**) into the extrusion droplets. All prepared protocells exhibited sufficiently strong adhesion, enabling the prototissues to withstand needle-

induced disturbances without disintegration. Scale bar, 1 cm. Experiments were repeated independently 3 times with similar results.

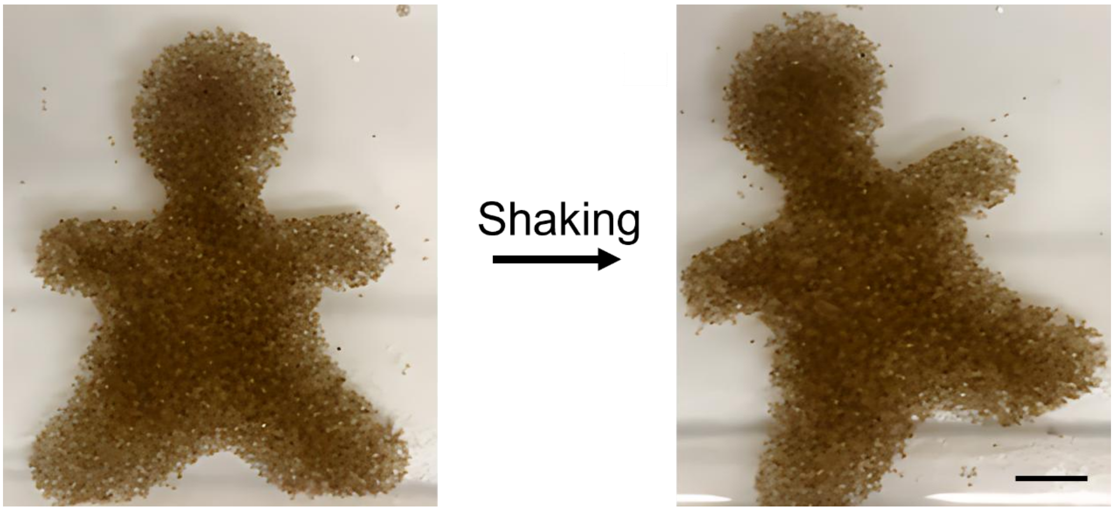

Supplementary Fig. 34: Structural stability of a humanoid-like prototissue under vigorous shaking. Digital images showing the structural stability of a humanoid-like prototissue under vigorous shaking. Scale bar, 1 cm. Experiments were repeated independently 3 times with similar results.

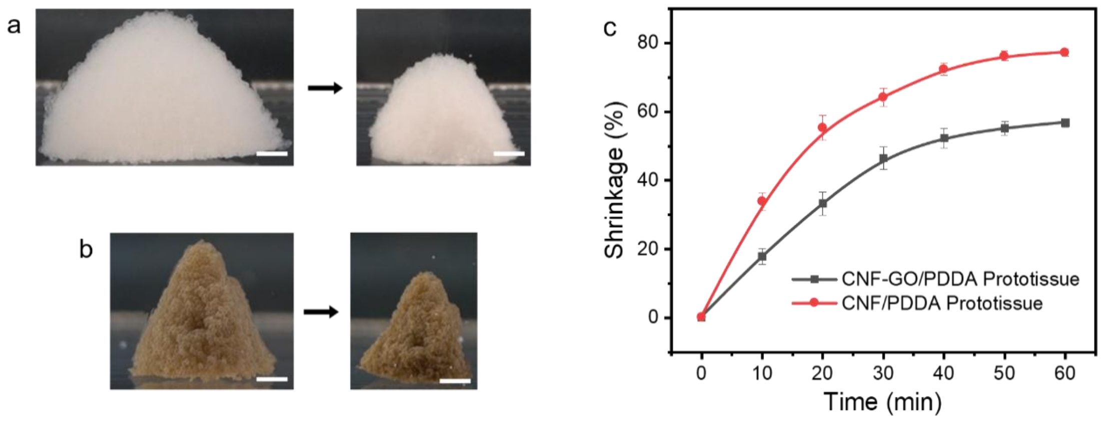

Supplementary Fig. 35: Osmotic shrinkage of CNF/PDDA and CNF-GO/PDDA prototissues. a, Side-view images of a CNF/PDDA prototissue before (left) and after 60 min immersion in 1 wt% PDDA-100k solution (right). b, Side-view images of a CNF–GO/PDDA prototissue before (left) and after 60 min immersion in 1 wt% PDDA-100k solution (right). c, Time evolution of the prototissue shrinkage for the two

compositions. Data are presented as mean values ± standard deviation (n = 3 independent experiments). Scale bar, 5 mm. Source data are provided as a Source Data file.

To quantitatively compare the mechanical strength of CNF/PDDA and CNF–GO/PDDA prototissues, we separately prepared conical CNF/PDDA and CNF–GO/PDDA prototissues and immersed them in a 1 wt% PDDA-100k aqueous solution. The time-dependent volume shrinkage of each prototissue was recorded and quantified as the volume shrinkage ratio, calculated as $1 - V/V_0$ based on the cone volume before and after shrinkage. The CNF/PDDA prototissue exhibited both a faster and larger volume reduction than the CNF–GO/PDDA prototissue. After 1 h, the volume shrinkage ratios of the CNF/PDDA and CNF–GO/PDDA prototissues reached 77% and 56%, respectively. These results indicated that CNF/PDDA prototissues possessed lower mechanical strength and were more susceptible to osmotic-pressure-induced shrinkage.

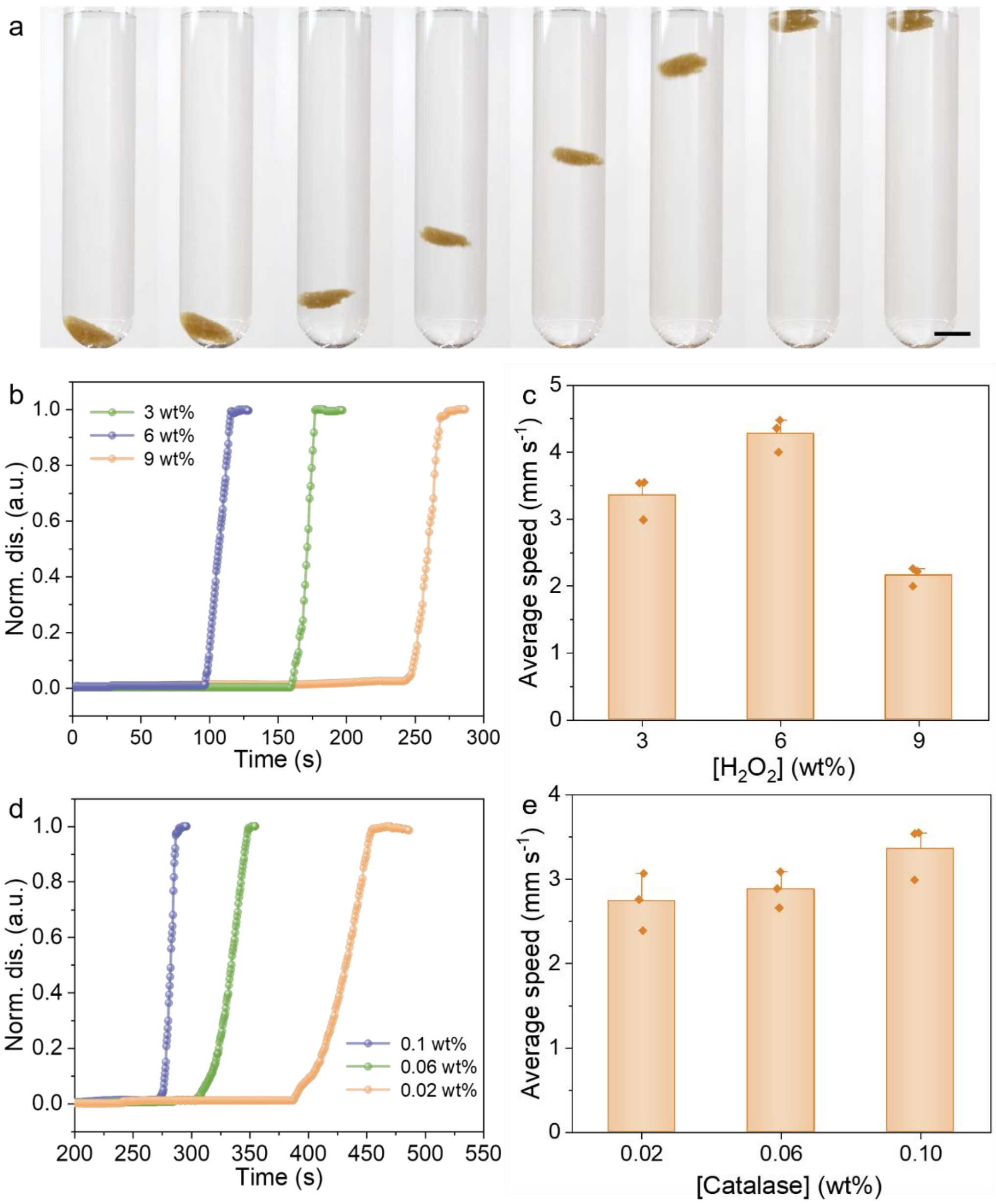

Supplementary Fig. 36: Buoyancy behavior of catalase-loaded CNF-GO/PDDA prototissues. a, Digital images showing a catalase-loaded CNF–GO/PDDA prototissue rising in aqueous solution containing H_2O_2 after an induction period. Scale bar, 1 cm. b, Height–time traces for prototissues with fixed catalase content (0.1 wt%) in H_2O_2 solutions of different concentrations (3, 6 and 9 wt%). c, Corresponding buoyancy rates, defined as the vertical ascent velocity obtained from the slope of the approximately linear rising segment of the curves in b. d, Normalized distance–time traces for prototissues with different catalase loadings at a fixed H_2O_2 concentration (3 wt%). e, Buoyancy rates as a function of catalase loading. Data are presented as mean values ±

standard deviation (n = 3 independent experiments). Source data are provided as a Source Data file.

The buoyancy rate was defined as the vertical ascent velocity of the prototissue, obtained from the slope of the height-time curves during the approximately linear rising stage after the induction period. When the catalase content in the CNF–GO/PDDA prototissue was fixed at 0.1 wt%, increasing the H_2O_2 concentration from 3 wt% to 6 wt% accelerated bubble generation and led to a higher ascent velocity, whereas further increasing the H_2O_2 concentration to 9 wt% slowed down the motion because of partial inhibition of catalase at excessive substrate levels. On the other hand, when the external H_2O_2 concentration was fixed at 3 wt%, reducing the amount of encapsulated catalase led to a systematic decrease in the buoyancy rate, thus the prototissue rose more slowly.

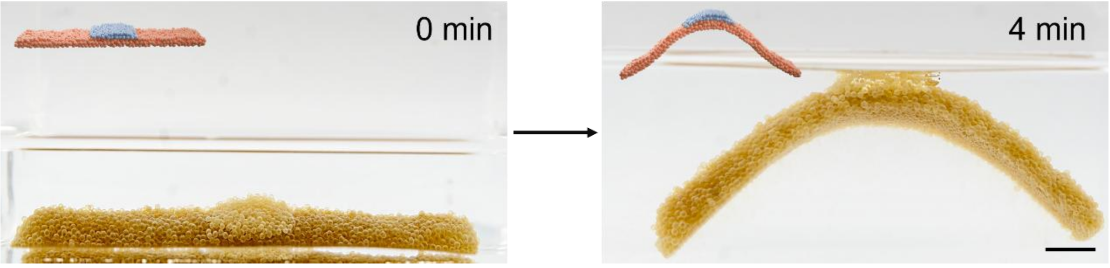

Supplementary Fig. 37: Deformation of a strip-shaped prototissue with localized catalase-loaded prototissues. Schematic illustration and digital images showing the deformation of a strip-shaped prototissue with catalase-loaded CNF-GO/PDDA prototissue deposited in the middle. Scale bar, 1 cm. Experiments were repeated independently 3 times with similar results.

Supplementary References

1. Wu, H., Du, X., Meng, X., Qiu, D. & Qiao, Y. A three-tiered colloidosomal microreactor for continuous flow catalysis. *Nat. Commun.* **12**, 6113 (2021).

2. Zhou, Y., Maitz, M. F., Zhang, K., Voit, B. & Appelhans, D. Dynamic and Diverse Coacervate Architectures by Controlled Demembranization. *J. Am. Chem. Soc.* **147**, 12239–12250 (2025).

3. Ji, Y., Lin, Y. & Qiao, Y. Interfacial assembly of biomimetic MOF-based porous membranes on coacervates to build complex protocells and prototissues. *Nat. Chem.* **17**, 986–996 (2025).

4. Zhou, Y. *et al.* Continuous Transformation from Membrane-Less Coacervates to Membranized Coacervates and Giant Vesicles: Toward Multicompartmental Protocells with Complex (Membrane) Architectures. *Angew. Chem. Int. Ed.* **63**, e202407472 (2024).

5. Ji, Y., Lin, Y. & Qiao, Y. Plant Cell-Inspired Membranization of Coacervate Protocells with a Structured Polysaccharide Layer. *J. Am. Chem. Soc.* **145**, 12576–12585 (2023).

6. Abdelhalim, A. O. E. *et al.* Reduction and functionalization of graphene oxide with L-cysteine: Synthesis, characterization and biocompatibility. *Nanomedicine Nanotechnol. Biol. Med.* **29**, 102284 (2020).
