# Supplementary material for: One-step construction of robust protocells and prototissues in water
Source: Nat Commun. 2026 Apr 8;17:4998. doi: 10.1038/s41467-026-71650-2 (PMC13237145; doi:10.1038/s41467-026-71650-2)
Supplement: Supplementary file 2 — Description of Additional Supplementary Files [file 41467_2026_71650_MOESM2_ESM.pdf]

Description of Additional Supplementary Files

Supplementary Movie 1: Fabrication of CNF/PDDA microcapsules via gas-liquid microfluidic-assisted DIC

Supplementary Movie 2: Unsuccessful fabrication of CNF/PDDA microcapsules without Triton X-100

Supplementary Movie 3: Fabrication of CNF-GO/PDDA microcapsules via gas-liquid microfluidic-assisted DIC

Supplementary Movie 4: Construction of a CNFGO/PDDA prototissue via gasliquid microfluidic-assisted DIC

Supplementary Movie 5: Osmotic pressure-induced shrinkage/swelling of a CNFGO/PDDA prototissue

Supplementary Movie 6: Construction of a humanoid-like CNF-GO/PDDA prototissue via molding

Supplementary Movie 7: Mechanical stability of a humanoid-like CNF-GO/PDDA prototissue

Supplementary Movie 8: Osmotic pressure-induced deformation of a Janus starfishlike prototissue

Supplementary Movie 9: Buoyancy-driven motion of a catalase-loaded CNFGO/PDDA protocell

Supplementary Movie 10: Buoyancy-driven motion of a catalase-loaded CNFGO/PDDA prototissue

Supplementary Movie 11: Buoyancy-driven motion/deformation of a stripshaped CNF-GO/PDDA prototissue I

Supplementary Movie 12: Buoyancy-driven motion/deformation of a stripshaped CNF-GO/PDDA prototissue II

Supplementary Movie 13: Buoyancy-driven motion/deformation of a stripshaped CNF-GO/PDDA prototissue III

Supplementary Movie 14: Buoyancy-driven motion/deformation of a stripshaped CNF-GO/PDDA prototissue IV

Supplementary Movie 15: Buoyancy-driven motion/deformation of a crossshaped CNF-GO/PDDA prototissue

Supplementary Movie 16: Soft gripper based on a crossshaped CNF-GO/PDDA prototissue

Supplementary Movie 17: Vertical oscillatory motion of a catalase-loaded CNFGO/PDDA prototissue
