# Supplementary material for: One-step construction of robust protocells and prototissues in water
Source: Nat Commun. 2026 Apr 8;17:4998. doi: 10.1038/s41467-026-71650-2 (PMC13237145; doi:10.1038/s41467-026-71650-2)
Supplement: Supplementary file 20 — Reporting Summary [file 41467_2026_71650_MOESM20_ESM.pdf]

nature portfolio

Corresponding author(s): Shaowei Shi, Fu-Jian Xu

Last updated by author(s): Mar 22, 2026

Reporting Summary

Nature Portfolio wishes to improve the reproducibility of the work that we publish. This form provides structure for consistency and transparency in reporting. For further information on Nature Portfolio policies, see our Editorial Policies and the Editorial Policy Checklist.

Statistics

For all statistical analyses, confirm that the following items are present in the figure legend, table legend, main text, or Methods section.

n/a	Confirmed	
☐	☒	The exact sample size (*n*) for each experimental group/condition, given as a discrete number and unit of measurement
☐	☒	A statement on whether measurements were taken from distinct samples or whether the same sample was measured repeatedly
☐	☒	The statistical test(s) used AND whether they are one- or two-sided<br>*Only common tests should be described solely by name; describe more complex techniques in the Methods section.*
☒	☐	A description of all covariates tested
☐	☒	A description of any assumptions or corrections, such as tests of normality and adjustment for multiple comparisons
☐	☒	A full description of the statistical parameters including central tendency (e.g. means) or other basic estimates (e.g. regression coefficient) AND variation (e.g. standard deviation) or associated estimates of uncertainty (e.g. confidence intervals)
☐	☒	For null hypothesis testing, the test statistic (e.g. *F*, *t*, *r*) with confidence intervals, effect sizes, degrees of freedom and *P* value noted<br>*Give P values as exact values whenever suitable.*
☒	☐	For Bayesian analysis, information on the choice of priors and Markov chain Monte Carlo settings
☒	☐	For hierarchical and complex designs, identification of the appropriate level for tests and full reporting of outcomes
☒	☐	Estimates of effect sizes (e.g. Cohen's *d*, Pearson's *r*), indicating how they were calculated

Our web collection on statistics for biologists contains articles on many of the points above.

Software and code

Policy information about availability of computer code

Data collection	Data were collected using instrument control and acquisition software associated with the ZEISS Imager.A2 polarized optical microscope (ZEISS ZEN, version 3.7), JEOL JSM-7900F SEM, Bruker FastScan DMFASTSCAN2-SYS AFM (Bruker NanoScope software, version 8.x), Leica TCS SP8 laser scanning confocal microscope attached to a Leica DMi8 inverted fluorescence microscope (Leica Application Suite X (LAS X), version 3.7.3, including the FRAP module), Thermo Scientific Nicolet 6700 FTIR spectrometer, Renishaw inVia Reflex Raman microscope (WiRE software), Malvern Zetasizer Nano ZS90 DLS instrument (Zetasizer software, version 8.02), and Thermo Scientific ESCALAB 250 XPS system (Avantage, version 5).
Data analysis	Data analysis was performed using ImageJ (NIH, version 1.54f), Origin/OriginPro (OriginLab, version 2018) and Microsoft Excel (Microsoft 2021). Confocal image processing and fluorescence intensity quantification were performed using Leica Application Suite X (LAS X, Leica, version 3.7.3). XPS spectra were fitted using XPSPEAK. AFM data were analyzed using Bruker NanoScope Analysis software (NanoScope Analysis 2.0).

For manuscripts utilizing custom algorithms or software that are central to the research but not yet described in published literature, software must be made available to editors and reviewers. We strongly encourage code deposition in a community repository (e.g. GitHub). See the Nature Portfolio guidelines for submitting code & software for further information.

Data

Policy information about availability of data

All manuscripts must include a data availability statement. This statement should provide the following information, where applicable:

- Accession codes, unique identifiers, or web links for publicly available datasets
- A description of any restrictions on data availability
- For clinical datasets or third party data, please ensure that the statement adheres to our policy

Data supporting the findings of this study are available within the paper, its Supplementary Information files and from corresponding authors upon request. Source data for all figures in the main text and Supplementary Information are provided with this paper.

Research involving human participants, their data, or biological material

Policy information about studies with human participants or human data. See also policy information about sex, gender (identity/presentation), and sexual orientation and race, ethnicity and racism.

Reporting on sex and gender	Not applicable. This study did not involve human participants, human data, or human biological material.
Reporting on race, ethnicity, or other socially relevant groupings	Not applicable. This study did not involve human participants, human data, or human biological material.
Population characteristics	Not applicable. This study did not involve human participants, human data, or human biological material.
Recruitment	Not applicable. This study did not involve human participants, human data, or human biological material.
Ethics oversight	Not applicable. This study did not involve human participants, human data, or human biological material.

Note that full information on the approval of the study protocol must also be provided in the manuscript.

Field-specific reporting

Please select the one below that is the best fit for your research. If you are not sure, read the appropriate sections before making your selection.

☒ Life sciences ☐ Behavioural & social sciences ☐ Ecological, evolutionary & environmental sciences

For a reference copy of the document with all sections, see nature.com/documents/nr-reporting-summary-flat.pdf

Life sciences study design

All studies must disclose on these points even when the disclosure is negative.

Sample size	Sample sizes were not predetermined by statistical power analysis. Sample sizes were chosen based on prior experience in similar experiments and pilot measurements to ensure reproducibility.
Data exclusions	No data were excluded from the analyses.
Replication	Key experiments were independently repeated 3 times with similar results.
Randomization	Randomization was not applicable to this study because the experiments involved in vitro material systems and instrument-based measurements rather than allocation of participants/organisms to treatment groups. Comparisons between conditions were performed under otherwise identical experimental settings.
Blinding	Blinding was not applicable because the study did not involve subjective outcome assessment or participant allocation. Data acquisition was instrument-based, and image/data analyses followed predefined and consistent procedures to minimize bias.

Reporting for specific materials, systems and methods

We require information from authors about some types of materials, experimental systems and methods used in many studies. Here, indicate whether each material, system or method listed is relevant to your study. If you are not sure if a list item applies to your research, read the appropriate section before selecting a response.

Materials & experimental systems

n/a	Involved in the study	
☒	☐	Antibodies
☐	☒	Eukaryotic cell lines
☒	☐	Palaeontology and archaeology
☒	☐	Animals and other organisms
☒	☐	Clinical data
☒	☐	Dual use research of concern
☒	☐	Plants

Methods

n/a	Involved in the study	
☒	☐	ChIP-seq
☒	☐	Flow cytometry
☒	☐	MRI-based neuroimaging

Eukaryotic cell lines

Policy information about cell lines and Sex and Gender in Research

Cell line source(s)	The murine fibroblast L929 cell line was obtained from the Cell Bank of the Chinese Academy of Sciences. These cells are originally derived from a male C3H mouse.
Authentication	The L929 cell line was authenticated by the vendor via STR profiling. No further authentication was performed in our laboratory.
Mycoplasma contamination	All cell lines tested negative for mycoplasma contamination.
Commonly misidentified lines (See ICLAC register)	No commonly misidentified cell lines from the ICLAC register were used in this study.

Plants

Seed stocks	Not applicable
Novel plant genotypes	Not applicable
Authentication	Not applicable
